# Experimental performance, exergy, and economic analysis of an oval tubular solar still integrated with nano-enhanced phase change material

**DOI:** 10.1038/s41598-026-43990-y

**Published:** 2026-04-02

**Authors:** Wael I. A. Aly, Mostafa A. Tolba, Mahmoud Abdelmagied

**Affiliations:** Department of Refrigeration and A/C Dpt., Faculty of Technology and Education, Capital University (Formerly Helwan), 11282 Cairo, Egypt

**Keywords:** Solar desalination, Oval tubular solar still, Productivity enhancement, Exergy efficiency, Paraffin wax, Nano alumina Paraffin wax, Energy science and technology, Engineering, Environmental sciences, Materials science

## Abstract

Freshwater scarcity remains a major challenge in arid and coastal regions, and conventional solar stills often suffer from low productivity, limited thermal efficiency, and high operational costs, highlighting the need for improved designs. This work examines a new form of the oval tubular solar stills, OTSS. This study focuses on enhancing the daily, nighttime, and overall (diurnal) productivity of freshwater by employing advanced materials. In the present investigation, paraffin wax (PW) was used as a phase change material (PCM), while nano-alumina-enhanced paraffin wax (NAPW) was applied to improve the thermal conductivity of PW. The OTSS is a simply distinguish design, lower cost, and high production rate of desalinated water. The OTSS is used with PW, without PW, and with NAPW. The experiments were conducted under Cairo, Egypt climatic conditions (Latitude 30.10 N longitude 31.29E) with varying basin water depths (0.5–2 cm) and Al_2_O_3_ concentrations (0.1–0.3 wt%). Results show that OTSS with PW achieved a maximum productivity of 6.53 L/m^2^/day, while the addition of NAPW further increased productivity to 7.26 L/m^2^/day and thermal efficiency to 68.24%. Daily thermal exergy efficiency improved from 3.41% (without PW) to 4.52% (with NAPW), and the production cost decreased from $0.0208 to $0.0163 per liter. The study demonstrates the effectiveness of integrating PCM and nanoparticles in OTSS to improve freshwater yield, thermal performance, and economic feasibility.

## Introduction

Water is the most important demand of life on the surface of the earth, and although two-thirds of the earth is surrounded by water, only 3% of all water sources are drinkable, and the rest is salty water. With the increase in the population every day, this small amount of water becomes insufficient to meet the needs of this population increase. Thus, the ideal solution is to remove salts from salt water to obtain fresh water suitable for drinking^1–7^. Water treatment processes to remove salinity are carried out using various methods, some of which have been known for centuries, while others are modern. One of the most widely used methods, employed for several centuries, involves evaporating water and then condensing it on cold surfaces. This principle forms the basis of the solar still, which has gained widespread use in recent times^8–11^.

Which has spread widely in recent times. Solar stills are highly suitable for small- to medium-scale applications in dry, arid, and coastal regions. These areas often lack adequate energy sources for desalination and the necessary infrastructure for transporting and storing desalinated water, resulting in water scarcity^12^. Despite the increase in the number of researches and researchers on solar distillates, the daily production of desalinated water is limited to 2.50 L/m^2^/day^13^. As well as the low-efficiency value, which is about 30%^14^. Because of the low productivity and also the low efficiency of this device, many studies achieved many experiments to enhancement the solar stills performance and the daily productivity^15^. The change of the outer shape design of the solar stills, like the pyramid solar still, achieved productivity of 3.5 L/m^2^/day^16–20^. The rectangular solar still design with a phase change material present 1.6 L/m^2^/day productivity and the improvement rate after using the phase change material was 52%^21^. The spherical solar still, which was implemented in India, had a 4.2 L/m^2^/day in productivity and the 42% in the efficiency^22^. The circular solar still had a 4.51 L/m^2^/day productivity without any additions to improve performance^8^. Some other researchers tended to add materials or parts that improve the performance of solar stills, such as using hot water heated by solar radiation ^1–24^, the use of nanofluids, which achieved a 38% increase in productivity^25,26^, use an air compressor to pump hot air into the saltwater basin^27^, the use of phase change^28^ , the solar chimney led to 46.3% increases in the productivity^29^, Installing inner and outer condensers^30,31^, paint the basin in black with using nanomaterial^32^, blended wick and cowl cooling^33^, the combination of PCM and nanoparticles, beside the using of PW with Al_2_O_3_ nanoparticles, the performance improves by 45%^24^.

Also,^34^ presented experimental studies on a solar still under three different configurations: the first case was a conventional solar still without any additives; the second case involved the addition of a PCM; and the third case integrated nanoparticles with the PCM. The results showed an improvement in productivity by 92% after adding only phase change material in the second case and got an improvement of 106% after combining the nanoparticles with the phase change material in the third case compared to the traditional first case. Also,^35^ experimental studies were presented for a pyramid solar still using three nanofluids (AlO, SnO_2_, and ZnO) and it achieved an improvement in productivity by (29.59, 18.63, and 12.67) %, respectively. He also presented^36^ experimental research for a hierarchical solar distiller that combines the PCM, PW, and the graphite nanoparticle. The productivity reached 8.45 L/m^2^/day of the system, and the improvement was 65.13% compared to the traditional case. The addition of nanoparticles to the solar still has a significant and interesting positive effect, and they confirmed that we need more research on this point^37,38^. An experimental work to present the pyramidal solar distiller with comparing three nanoparticles (Al_2_O_3_, CuO, and TiO_2_) with saline water was achieved^39,40^. The results showed the superiority of water/Al_2_O_3_ over (water/CuO, and water/TiO_2_).

In the year (2021), presented an experimental study of two models of pyramid solar stills single-slope under the climatic condition of Alexandria—Egypt. Copper oxide (CuO) at different concentrations (0.5, 1, and 1.5) % was used at different depths of saline water in the basin (1, 2, and 3) cm and it was found that the best performance of the solar still was at the lowest water depth of 1 cm and the highest concentration of nanoparticles 1.5% using the cooling cover and the vibrations resulting from the motor, and the system productivity was 7.13 L/m^2^/day and the efficiency increased by 54%, and the cost was 0.0276 $/ L/m^2^ of freshwater^1^. Also, in 2021, an experimental study was conducted in India for a pyramid single-slope solar still with the aim of increasing the productivity by paint the salt water basin with black paint mixed with nano (SiO_2_) and the results achieved an increase in productivity by 8.78% at a depth of 15 mm of saltwater in the basin^41^.

An experimental was given under Tanta city-Egypt weather conditions. Without PW, the system’s productivity and efficiency were 4.31 L/m^2^/day and 33.8%, respectively; however, with PW added, the productivity and efficiency rose to 9.05 L/m^2^/day and 72.7%, respectively, with a productivity improvement rate of 115%^42^. An experimental study was carried out under three cases of circular tubular solar still of (traditional PCM with PW, and PW with graphene nanoparticles). The productivity was 4.3, 6, and 7.9 L/m^2^ respectively, for the three cases and efficiency reach 31, 46, and 59%,^43^. The use of locally available gravel in cylindrical shape water desalination in Saudi Arabia was experimental achieved. The system used as a means to store heat. The efficiency and productivity reached to 31.9% and 3.96 L/m^2^/day for the xase of without adding gravel and 36.34% and 4.51 L/m^2^/day for the case of adding gravel, respectively^22^. Also, in 2021, an experimental study was conducted in Iran with the aim of increasing productivity by using paraffin wax and using a cover cooling with different water cooling rates 0.7, 1.3, and1.8 L/min, and the results achieved an increase in system productivity by 15% at a flow rate of 1.3 L/min. The proportion of the freshwater generated from the saltwater entering the system was calculated, and its value was 70%^44^. Several studies to expect the performance of a solar still using various composite materials, porous absorbing materials, internal reflectors and woven wire mesh, steel wool pads and phase change materials integrated with Peltier modules and Heaters were presented by Abdelgaleel et al.^45–49^. The results showed that the still performance was enhanced by using reflectors, woven wire mesh, PCM, Peltier and heaters. Also the cost per liter of the modified solar still was reduced. A numerical study was also conducted in Najaf city—Iraq (2019) for a single slope solar still with PCM and Al_2_O_3_. Incorporating PW with Al_2_O_3_ at a concentration of 3% enhance the productivity to 6 L/m^2^/day compared to the traditional system with 5 L/m^2^/day which means that it achieved an improvement in productivity by 20 and 12% for NPCM and PCM, respectively^12^. The pyramid solar still is experimental study of with three cases of traditional, with PW PCM, material, and combining of PW with graphene nanoparticles at concentrations of 0.2:0.6. The productivity was 4, 5, and 6.25 L/m^2^/day respectively in the three cases, and the highest values was achieved with PW and graphene particles at a concentration of 0.6%^50^. A numerical study was also conducted in 2018 for a pyramid single-slope solar still under the weather conditions of the city of Rabat—Morocco, the study used different quantities of paraffin wax 10, 15, 20, and 25 kg in addition to using different concentrations of nanoparticles Al_2_O_3_ 0.02, 0.1, and 0.2% The highest productivity of the system was 7.74 L/m^2^/day when using 20 kg of paraffin wax with 0.2% concentration of Al_2_O_3_ nanoparticles, and the improvement rate in productivity was 48%^51^. The storage of solar thermal energy based on Myo-Inositol using two types of nanoparticles Cou, Al_2_O_3_, and the results proved the superiority of Al_2_O_3_ in the storage of combined solar thermal energy^52^. Also^53^ in 2014 presented a study to test the ability of paraffin wax to store heat, Aluminum oxide was used with different concentrations 1, 2, 3, 4, and 5% to improve the performance of paraffin wax in heat storage and discharging the experiments proved that the best concentration of aluminum oxide is 2% .Although several studies have investigated solar stills, there remain limitations in achieving high freshwater productivity, thermal efficiency, and cost-effectiveness simultaneously. The novelty of the present work lies in the design and experimental investigation of a new OTSS integrated with PW as a PCM and NAPW to improve thermal conductivity. This study provides a comprehensive evaluation of the OTSS^8^ performance in terms of daily, nighttime, and overall (diurnal) water production, thermal exergy efficiency, and economic feasibility. The combined use of an innovative design with advanced thermal storage materials and nanoparticles has rarely been explored. This experimental study was conducted using saline water at basin depths ranging from 0.5 to 2 cm. A fixed mass of 1 kg of PW was employed to store solar thermal energy and release it after sunset, thereby extending operating hours and enhancing freshwater productivity. Additionally, Al_2_O_3_ nanoparticles at concentrations of 0.1–0.3 wt.% were integrated with the paraffin wax to further improve the system’s productivity.

### Novelty of the present study

The novelty of this work can be summarized as follows:

- A new oval tubular solar still (OTSS) design is experimentally proposed and evaluated, providing a larger condensation area compared to conventional solar stills.
- The combined integration of paraffin wax (PCM) and nano-alumina-enhanced paraffin wax (NAPW) is investigated within the OTSS, which has rarely been reported in previous studies.
- The effect of basin water depth on daily, nocturnal, and total freshwater productivity is systematically analyzed.
- Thermal exergy efficiency and economic performance are evaluated to provide a comprehensive performance assessment.

## Experimental setup and procedures

**Fig. 1:**
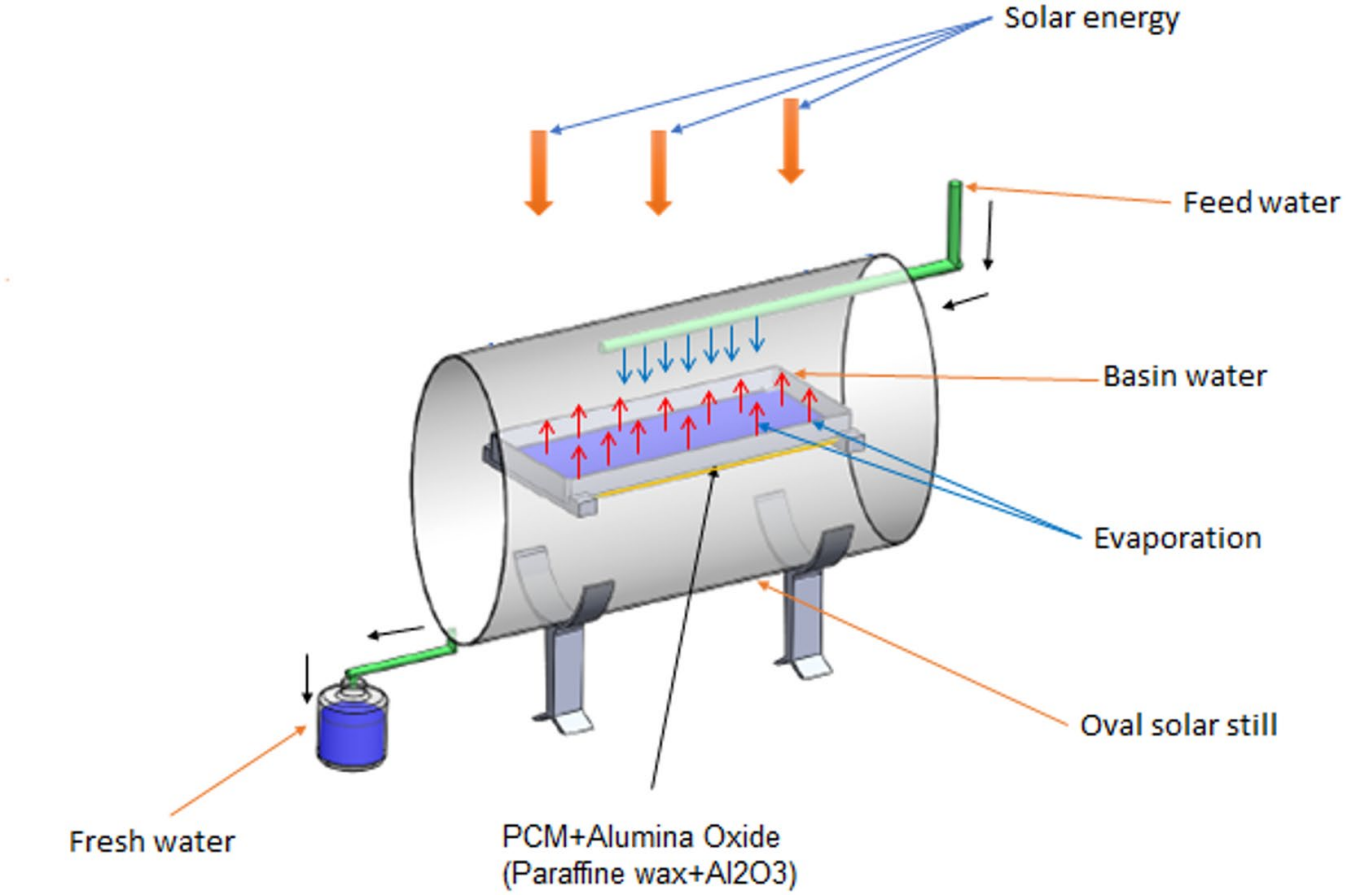
Schematic diagram of the experimental setup^8^.

**Fig. 2:**
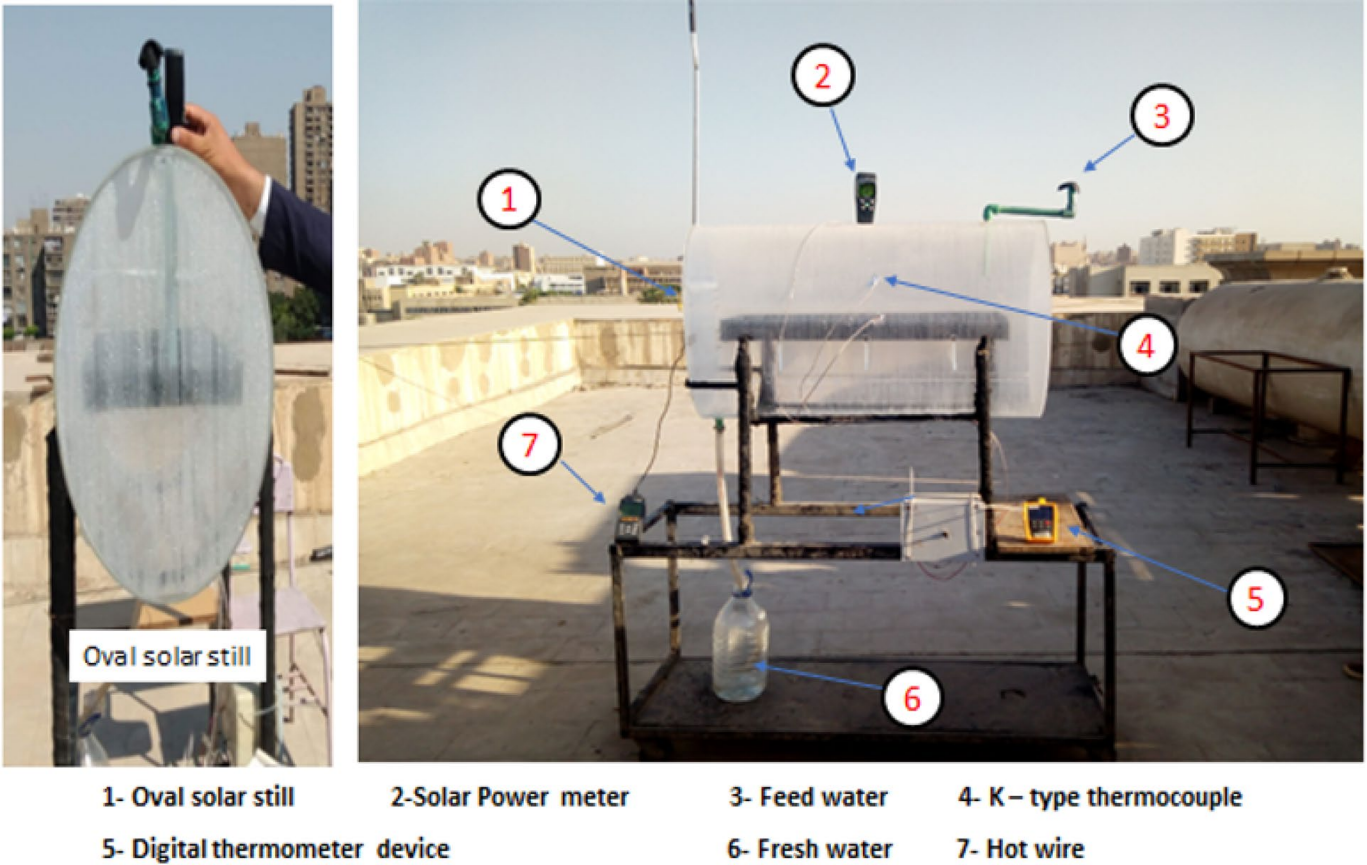
Photograph of the experimental setup of oval tubular solar still^8^.

The present study aims to enhance the performance of oval tubular solar stills (OTSS) using three approaches: (1) employing a new OTSS design, (2) integrating paraffin wax (PW) as a phase change material (PCM) to extend operating hours and productivity after sunset, and (3) incorporating Al_2_O_3_ nanoparticles with PW to further improve thermal performance. The OTSS (Figs. 1–2) is a transparent acrylic oval cylinder (Table 1 summarize the acrylic thermal properties), allowing sunlight penetration from all directions, which increases condensation surface area and heat transfer. The tube measures 100 cm in length, 60 cm in major diameter, 42 cm in minor diameter, and 5 mm in thickness. It contains a rectangular saltwater basin (80 × 30 × 5 cm) made of rust-resistant stainless steel painted black to enhance solar absorption. Saltwater in the basin evaporates under solar radiation, condenses on the inner oval surface, and is collected as freshwater at the bottom. Various basin water depths (0.5, 1, 1.5, and 2 cm) were tested to determine the optimum operating condition. The study uniquely examines how variations in Mediterranean Sea water (salinity 3009 ppm) influence real operating performance throughout daytime hours. Use a phase change material (paraffin wax) to store solar energy during the day as latent heat and release it after sunset. A two-floor basin is made of stainless steel. The paraffin wax was charged by 85% of the volume of the paraffin basin, because the volume of paraffin wax increases by 15% when changing from the solid-state to the liquid-state^42^.

**Table1:** Properties of the acrylic material^42^.

Property	Value	Unit
Thermal conductivity	0.19	W.m^−1^ .K^−1^
Heat capacity	1470	[J/kg ° K]
Forming temperature	170–190	[°C]
Density	1190	kg.m^−3^
Coefficient of thermal expansion	0.000072	K^−1^

**Fig. 3:**
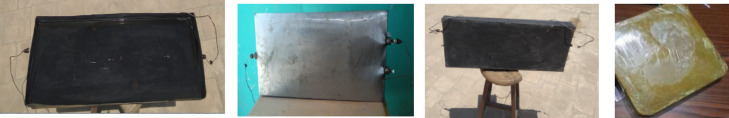
The shape of the water basin and paraffin wax plate.

**Fig. 4:**
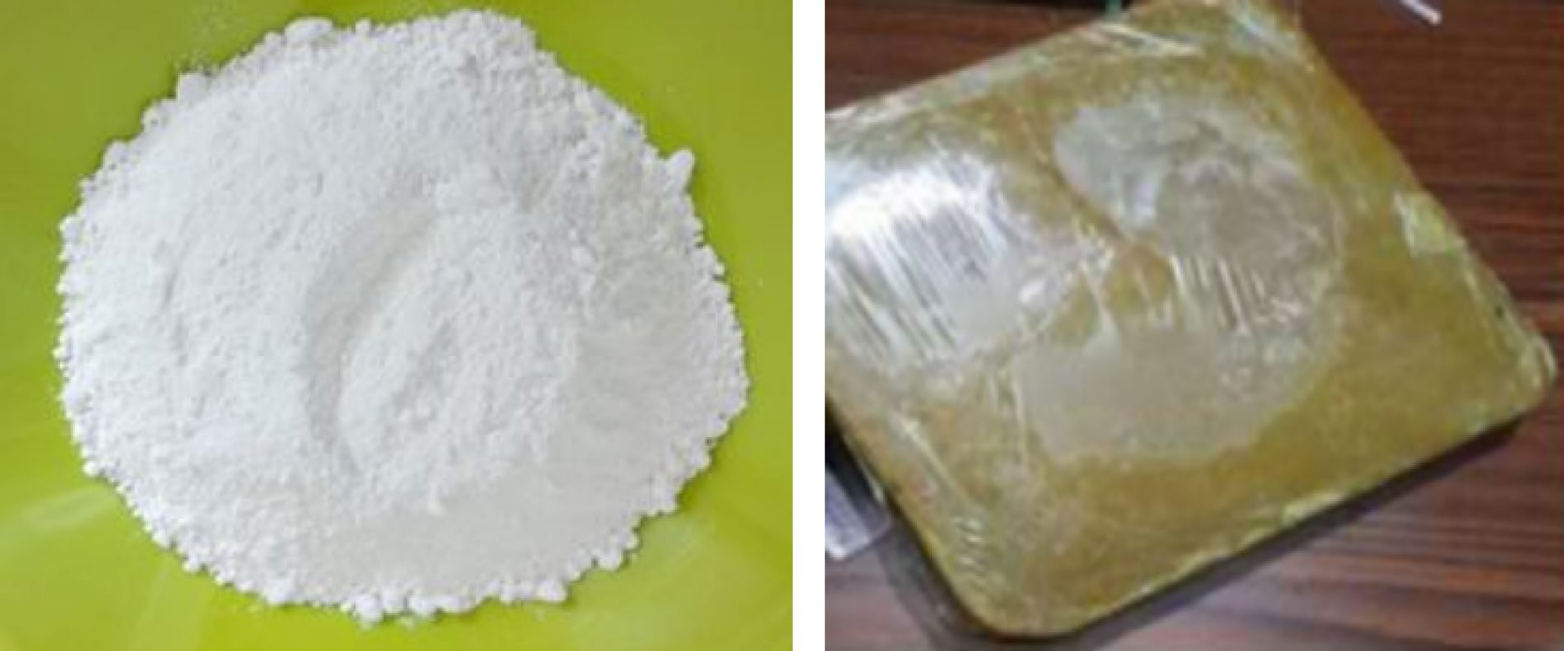
The working material (**A**) Al2O3 nanomaterial and (**B**) paraffin wax plate shape.

**Fig. 5:**
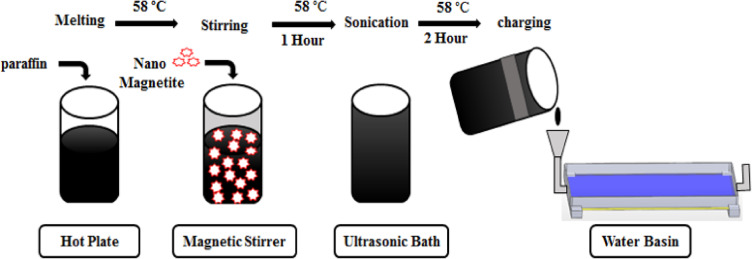
Steps of preparation of Al2O3/ PCM.

In the second approach, a two-layer stainless steel basin was used, with PW occupying 85% of the lower layer volume to accommodate thermal expansion during phase change. The upper layer contained saltwater. The system included ports and sensors for monitoring temperature. PW was selected for its local availability and reasonable cost (~ 4.4 USD/kg). Figure 3 and Table 2 show the basin configuration and PW properties. The third approach involved dispersing Al_2_O_3_ nanoparticles (0.1–0.3 wt.%) into PW (melting point 58 °C) to enhance heat transfer and OTSS yield after sunset. The nanofluid was mixed intermittently for two hours to avoid excessive temperatures. During operation, PW melts from late afternoon, releases heat after sunset, and solidifies overnight before returning to ambient temperature. Figures 4–5 and Table 3 illustrate the nanoparticles, nano-composite preparation, and their properties.

**Table 2:** The PCM (paraffin wax) thermal properties.

Property	Value	Unit
Peak melting temperature	57.23	[°C]
Peak solidification temperature	48.38	[°C]
Specific heat capacity	2.0	[KJ/ (kg. °C]
Density (solid)	880	[kg/m^3^ ]
Density (liquid)	760	[kg/m^3^ ]
Thermal conductivity (both phases)	0.2	[W/( m .°C) ]
Fusion Latent heat	90.7	[kJ/kg ]
Solidification Latent heat	78.2	[kJ/kg ]

**Table 3:** Properties of nanoparticles (Al_2_O_3_)^54,55^.

Property	Value	Unit
particle diameter	20	[nm]
Density	3880	[Kg/m^3^ ]
Conductivity	40	[W/ (m. K) ]
Specific heat capacity	773	[J/ (kg. K]

To determine the overall uncertainty associated with any derived function y, the following relation is applied:^16,56–58^.

1 $$w\left( u \right) = \sqrt {\left( {\frac{{\partial u}}{{\partial z_{1} }}} \right)^{2} m^{2} \left( {z_{1} } \right) + \left( {\frac{{\partial u}}{{\partial z_{2} }}} \right)^{2} m^{2} \left( {z_{2} } \right) + \ldots + \left( {\frac{{\partial u}}{{\partial z_{n} }}} \right)^{2} m^{2} \left( {z_{n} } \right)}$$

where ${\boldsymbol{w}}\left({\boldsymbol{u}}\right)$ the uncertainty of the variable *z*, m_*xn*_ is the uncertainty of parameter and z_*n*_ is the parameter of interest. Table 4 presents the measurement ranges, accuracies, errors, and uncertainties of the instruments employed in this study.

**Table 4:** Range, accuracy, and uncertainty of the measured and calculated parameters.

Instrument/Parameter	Range	Accuracy	Uncertainty
Thermocouples	−50 –1300 ˚C	± 0.1	± 0.10
Solar power meter	0–2000 W/m^2^	± 10	± 2.88
Calibrated flask	0–2000 mL	± 5	± 2.88
Hot wire anemometer	0.1–40 m/s	± 0.1	± 0.06

## Results and discussion

### Ambient and meteorological conditions

**Fig. 6:**
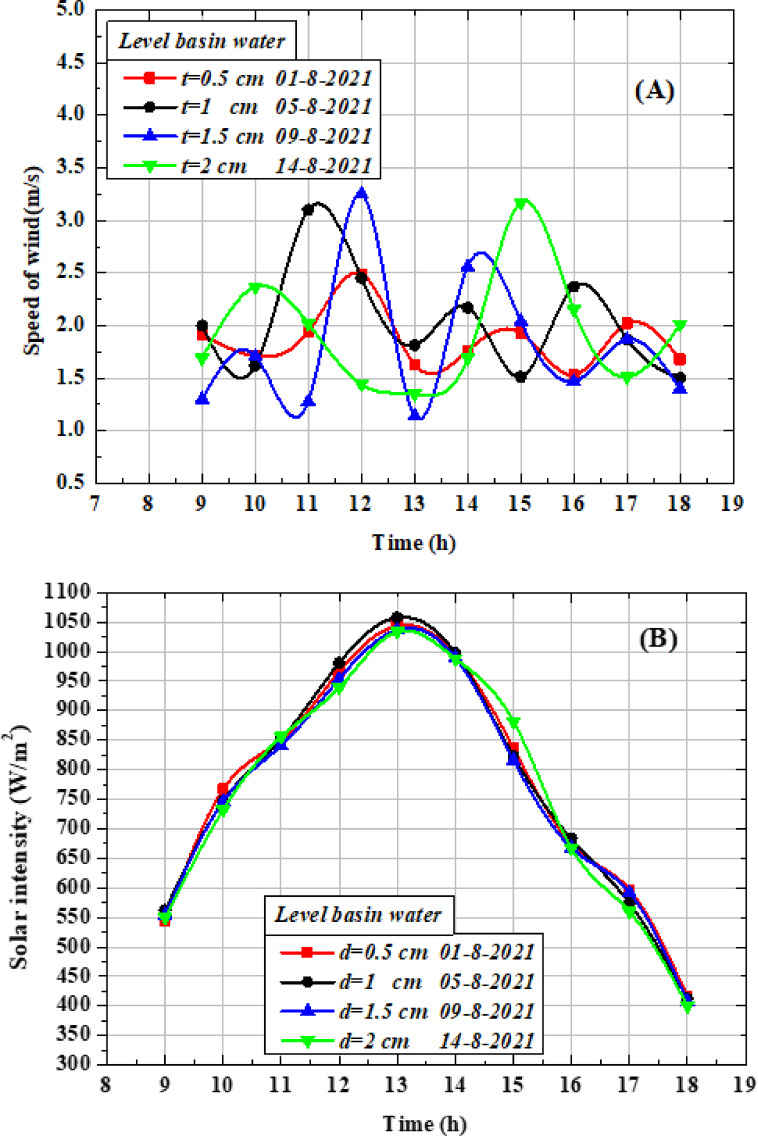

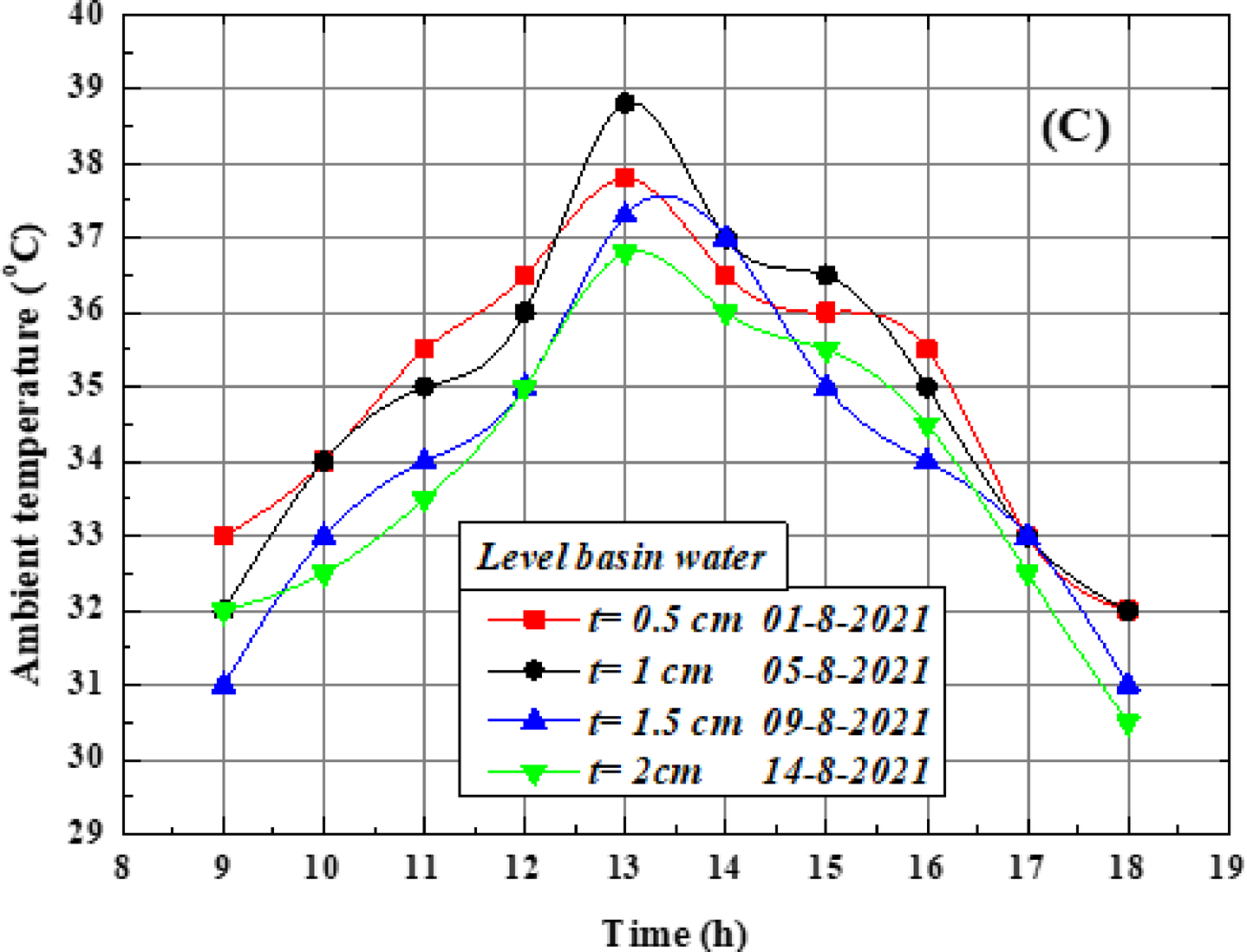
Climatic condition. (**A**) Speed of the wind, (**B**) solar intensity, and (**C**) ambient temperature.

The experimental weather conditions were characterized by the ambient temperature, solar radiation intensity, and wind speed, as these parameters directly influence the performance of the OTSS system. The tests were conducted during August 2021 on the rooftop of the Faculty of Technology and Education at Helwan University, Cairo, Egypt (latitude 30.10° N, longitude 31.29° E), under typical summer conditions. As an illustrative case, Fig. 6 presents the results for the system under the specific experimental conditions correspond to four representative days. The ambient temperature was observed to start at about 31 °C at the beginning of the test, increase to a peak of 38.8 °C around 13:00, and then gradually decline to nearly 30 °C after sunset Fig. 6(C). The solar radiation intensity, shown in Fig. 6(B), ranged from approximately 550 W/m^2^ at 9:00 a.m. to a maximum of 1058 W/m^2^ at noon, followed by a gradual reduction to nearly 400 W/m^2^ by 18:00. Meanwhile, wind speed variations during the experiments, recorded at different water depths in the basin Fig. 6(A), fluctuated between 1.14 m/s and 3.25 m/s.

### Effect of saltwater depth on OTSS performance

**Fig. 7:**
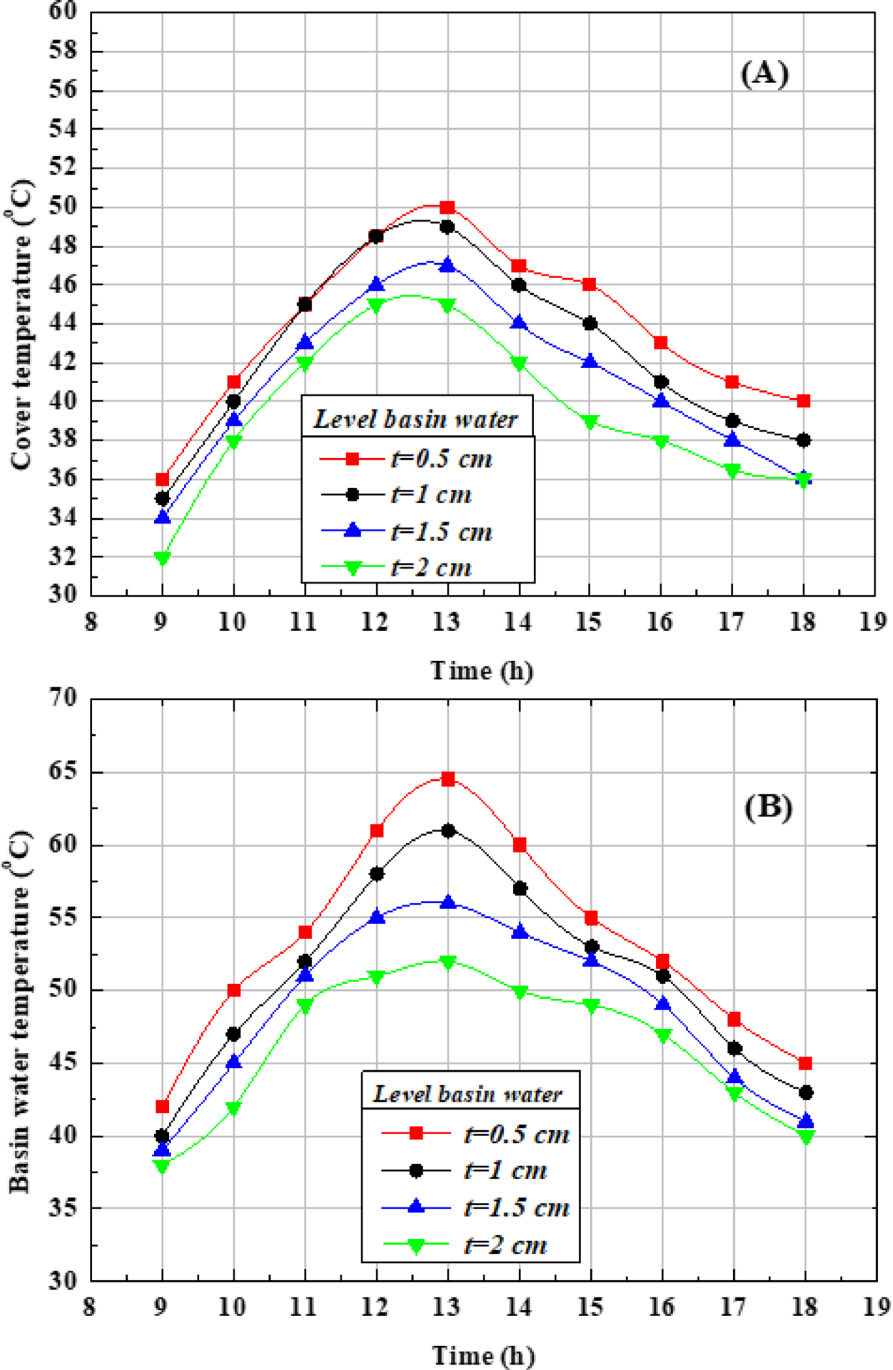
variation of basin saltwater depths on OTSS performance (**A**) temperature of the cover surface, and (**B**) temperature of the basin water.

The influence of varying saltwater depths in the basin was examined at four levels: 0.5, 1, 1.5, and 2 cm, to identify the optimum operating condition. Figure 7(a) shows the surface temperature of the solar still (polycarbonate) during the study days with different depths of water, where the lowest temperature of the oval tube surface reached 32°C and the maximum temperature 50°C. As seen the temperature difference between each of the four cases is relatively small compared to the difference in water temperatures. It is evident that reducing the saltwater depth in the basin accelerates the temperature rise of the water, which enhances the evaporation rate and consequently increases the cover temperature. In contrast, increasing the basin water depth results in lower cover temperatures. This indicates that the relationship between cover temperature and basin water depth is inverse, with the highest cover temperatures observed at the shallowest water depth. Figure 7(b) illustrates the variation of water temperature with time at these depths. It was observed that the lowest temperatures occurred at the start and end of the day (around 9:00 and 18:00), whereas the maximum temperature was recorded at midday (13:00). The highest value, 64.5 °C, was achieved at the shallowest depth of 0.5 cm at 1:00 p.m. For greater depths, the peak water temperatures were 61 °C at 1 cm, 56 °C at 1.5 cm, and 52 °C at 2 cm. These results indicate that shallower water depths heat up more rapidly, leading to higher evaporation rates. Conversely, larger water depths require more time to absorb heat, resulting in slower temperature rise and reduced evaporation.

**Fig. 8:**
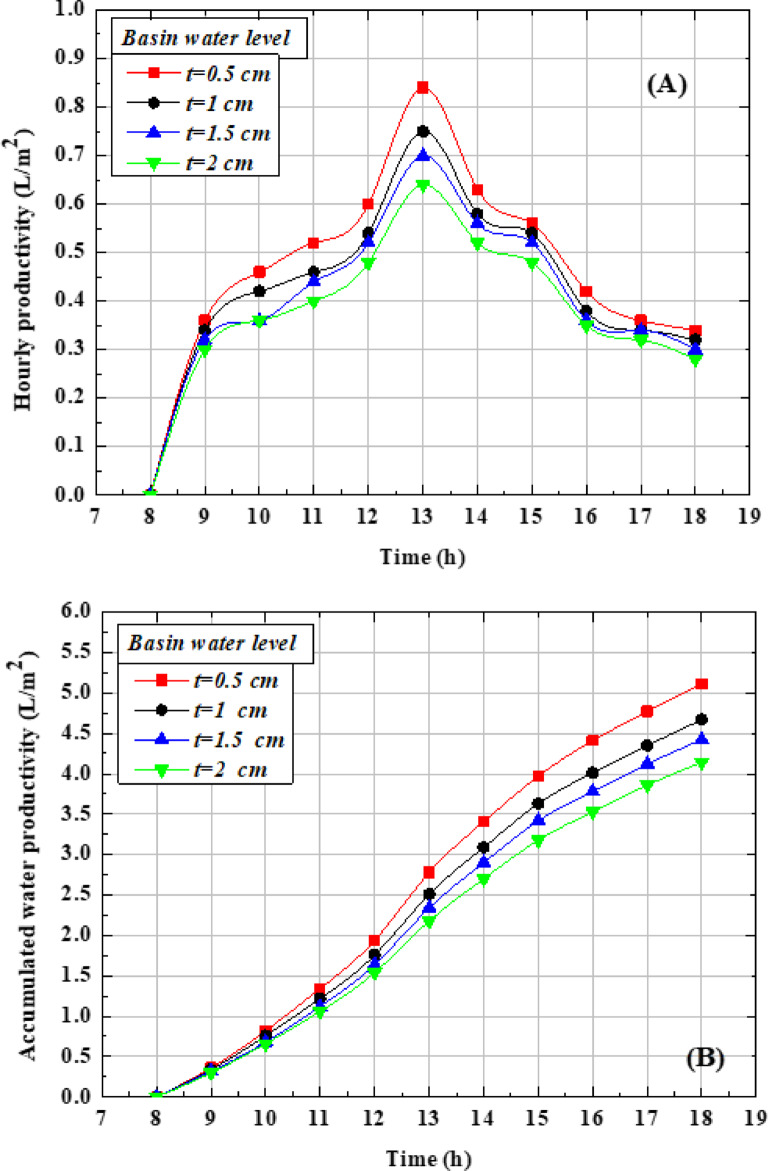
Effect of various saltwater depths on OTSS performance (**A**) hourly productivity (**B**) accumulated water productivity.

Figure 8(a) presents the hourly production of desalinated water at different basin depths. The output increases steadily from the morning hours, reaches its peak at midday, and then gradually declines after sunset. The maximum hourly yields recorded at water depths of 2, 1.5, 1, and 0.5 cm were 0.64, 0.70, 0.75, and 0.84 L/h, respectively. These results demonstrate that shallower water depths enhance the rate of freshwater production, as less heat and shorter heating time are required to initiate evaporation. Furthermore, the temperature difference between the tube surface and the saltwater becomes more pronounced as the water depth decreases, further contributing to higher evaporation and production rates. The smaller water depth presents the highest desalinated water production because less heat and time are required to raise the water temperature and achieve evaporation. Moreover, the temperature variation between the tube surface and the saltwater reaches its maximum when the water depth decreases^8^. Figure 8(b) shows the total daily freshwater production rates at different basin depths. The recorded values were 4.14, 4.42, 4.67, and 5.11 L/m^2^·day for water depths of 2, 1.5, 1, and 0.5 cm, respectively. These findings confirm that reducing the water depth enhances overall productivity. As seen, the accumulated production of desalinated water at the depth of 0.5 cm is higher than the depths of 2, 1.5, and 1 cm by 23.42%, 15.61%, and 9.42%, respectively.

### Effect of adding PCM on OTSS performance

**Fig. 9:**
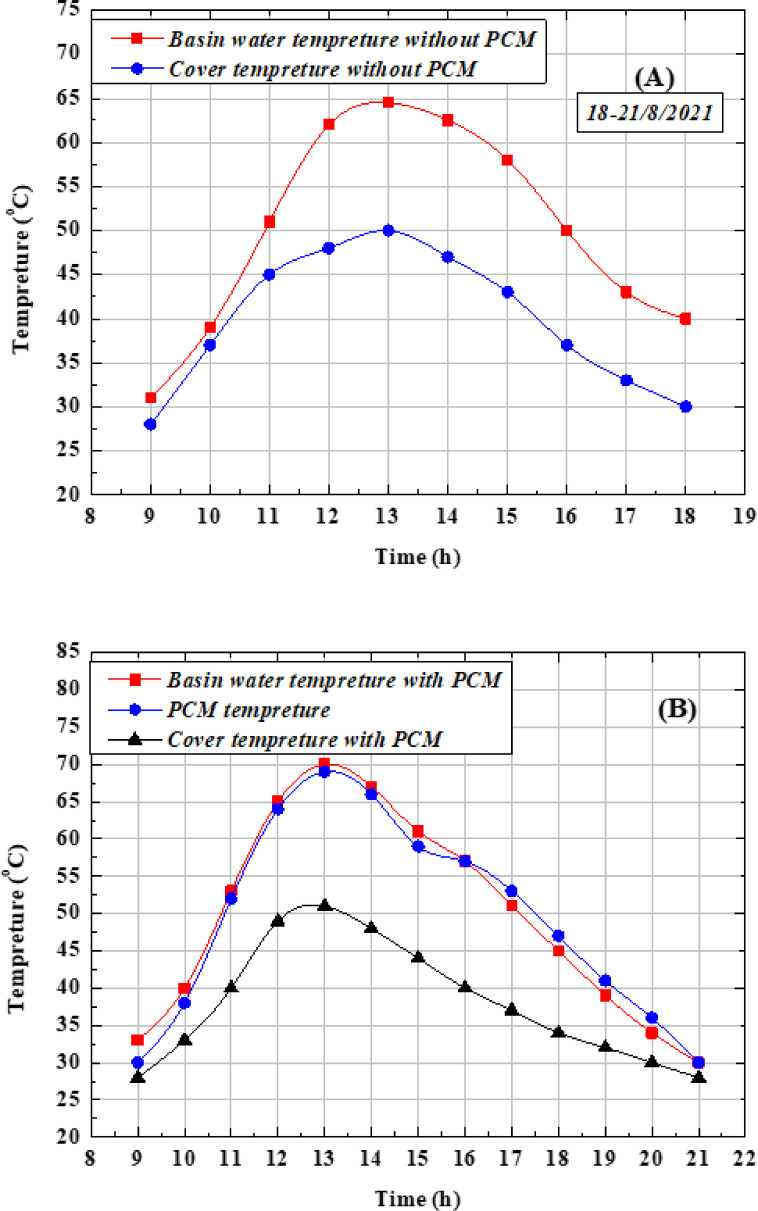
Climatic Temperature variation of PCM, basin water, and cover for (**A**) without PCM and (**B**) with PCM along the test day.

For this set of experiments, a saltwater depth of 0.5 cm inside the basin was selected. Figure 9(A) compares the basin water temperature with and without the use of PCM. The maximum temperature of the basin water with PCM reached 70 °C at 13:00, compared to 64.5 °C without PCM. Figure 9(b) presents the PCM temperature profile, which varied between 27 °C and 68 °C. The cover temperature ranged from 25–51 °C with PCM, while in the absence of PCM it fluctuated between 27–49 °C. In all cases, the temperatures of the monitored components increased gradually during the morning, reached their peak values around noon, and then decreased steadily afterward. The higher basin water temperature observed with PCM is attributed to the additional heat supplied by the material during periods of low solar intensity or after sunset. This process, known as the discharge phase, occurs when paraffin wax releases the stored heat. A similar trend has been reported in previous studies^56^. Furthermore, the decline in basin water temperature during the afternoon corresponds to the reduction in solar radiation. After approximately 16:00, the reheating effect of PCM becomes evident. Paraffin wax begins to melt in the first half of the day as its temperature rises with solar intensity until it reaches its melting point (≈57 °C). Complete melting occurs between 12:00 and 13:00, after which the material undergoes a phase change at nearly constant temperature while storing latent heat. Around 16:00, the discharging stage begins, during which the PCM gradually releases its stored heat until reaching the solidification point.

**Fig. 10:**
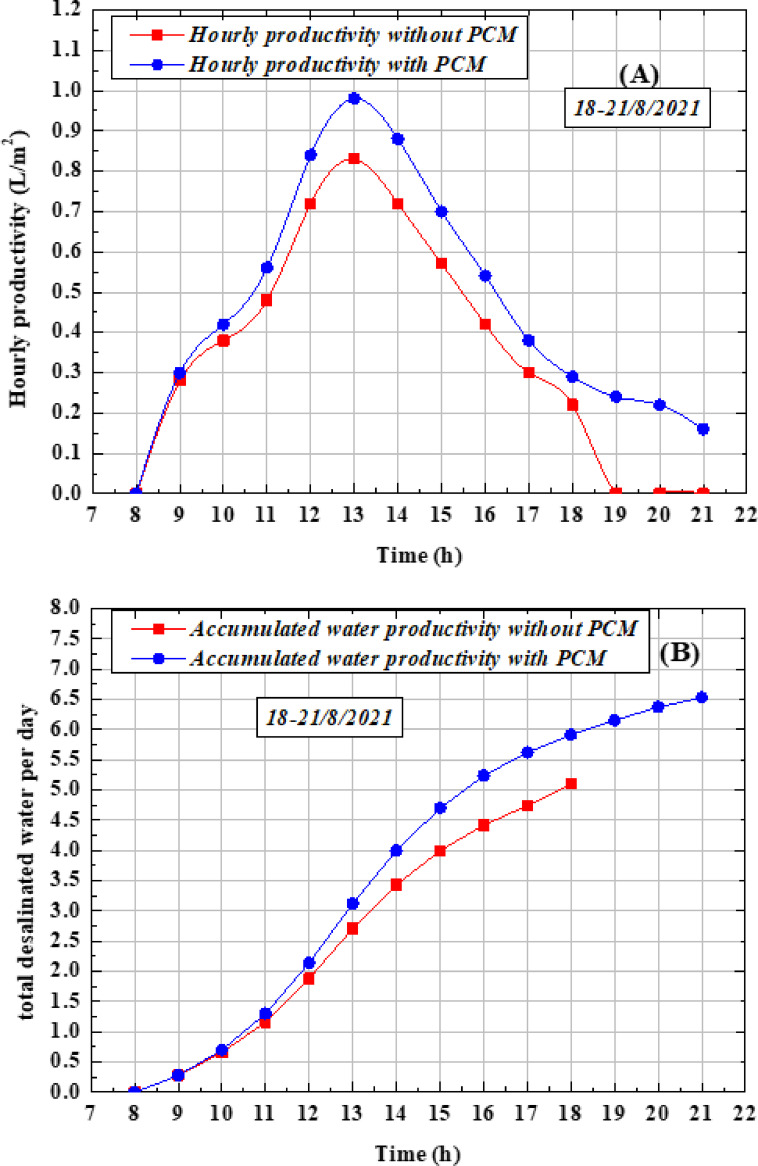
Performance of the OTSS with and without PCM (**a**) desalinated water production per hour (**b**) total desalinated water per day.

Figure 10(A) illustrates the hourly freshwater production between 8:00 a.m. and 9:00 p.m. with and without the use of PCM. The production rate starts from zero at 8:00 a.m., increases gradually, and reaches its maximum value around 13:00 at noon. The results clearly show that the incorporation of PCM enhances productivity compared to the system without PCM, particularly during the first half of the day (charging period). This behavior is attributed to both the high solar irradiance levels during this time and the suitable melting point of paraffin wax, which allows it to absorb and store heat. During the discharge period, when paraffin transitions from liquid to solid, the stored heat is released, providing an additional source of energy for the basin water during evening hours and periods of low solar intensity. This supplementary heating raises the water temperature, enhances the evaporation rate, and consequently increases freshwater productivity. The maximum hourly productivity achieved with PCM was 0.98 L/m^2^, compared to 0.84 L/m^2^ without PCM. Figure 10 (B) shows the cumulative freshwater output, where the daily yield with PCM reached 6.53 L/m^2^·day, while the corresponding value without PCM was 5.11 L/m^2^·day. This indicates that the use of PCM improved overall productivity by approximately 28.04%. Paraffin wax begins melting in the morning as solar irradiance increases, reaching full liquefaction around midday. In the afternoon, as solar intensity declines, the material gradually solidifies, releasing stored latent heat. This effect becomes particularly evident between 18:00 and 21:00, where an increase in water temperature and system output demonstrates the positive impact of PCM on extended operation.

### The effect of incorporating (Al_2_O_3_) with paraffin wax on OTSS performance

**Fig. 11:**
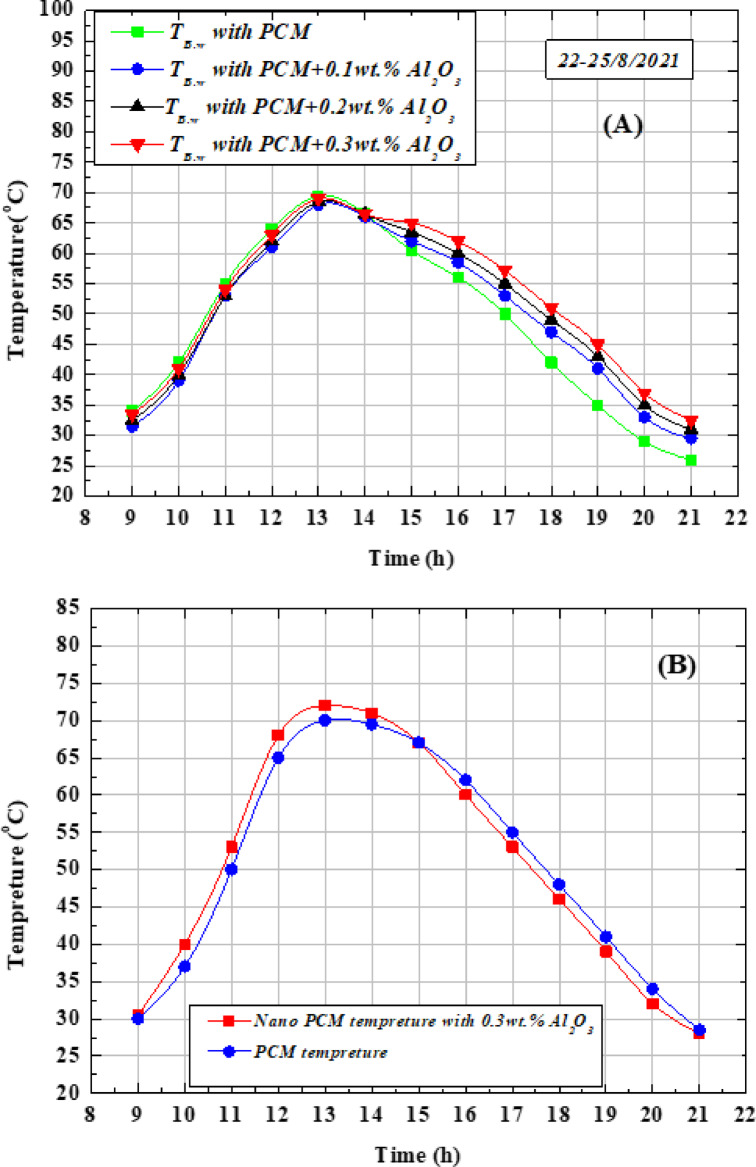
(**A**) Basin water temperature variation with PW and NAPW at different sizes concentrations of (Al2O3), and (**B**) Temperatures of paraffin wax (PW) and Al2O3-PW nanocomposite (NAPW) along the test day.

Figure 11(A) illustrates the variation of basin saltwater temperature using pure paraffin wax (PW) and paraffin wax enhanced with different concentrations of Al_2_O_3_ nanoparticles (NAPW) at ϕ = 0.1, 0.2, and 0.3 wt.%. The water temperature increased steadily, peaking around 1:00 pm, before gradually declining towards the evening. The maximum temperature with pure PW was about 70 °C, while the corresponding peak values with NAPW were 68, 68.5, and 69 °C, respectively. During the morning period (before 2:00 pm), the basin water with PW showed slightly higher temperatures compared to NAPW. This behavior is attributed to the enhanced thermal conductivity of NAPW, which promotes faster heat absorption and storage. However, after 2:00 pm, the NAPW samples exhibited higher temperatures, as the stored energy was released more effectively than with PW alone. In other words, the presence of Al_2_O_3_ nanoparticles accelerates heat transfer and enhances heat dissipation, resulting in improved basin water heating during the late afternoon and evening hours (2:00–9:00 pm). During the first half of the day (before 14:00), the basin water temperature was higher when using pure PW compared to NAPW. This behavior is linked to the enhanced thermal conductivity of NAPW, which enables faster heat absorption and storage, as confirmed by the results. In contrast, during the second half of the day (14:00–21:00), the basin water temperature with NAPW exceeded that of pure PW, owing to the accelerated release of the stored energy. In other words, NAPW exhibits faster charging during the morning hours and quicker discharging in the evening compared to pure PW. This trend is clearly illustrated in Fig. 11(A and B).

Figure 11(B) demonstrates that the temperature profile of NAPW is initially higher than that of pure PW during the first half of the day, whereas in the second half, the NAPW temperature becomes comparatively lower. This trend is mainly attributed to the enhanced thermal conductivity of NAPW, which increases from 0.200 W/m·°C for pure PW to 0.20314, 0.20628, and 0.20942 W/m·°C for NAPW at nanoparticle concentrations of 0.1, 0.2, and 0.3 wt. %, respectively. These values correspond to improvements of 1.57%, 3.14%, and 4.71% over PW. During the charging period (morning hours), the higher conductivity allows NAPW to absorb heat more rapidly than PW. Conversely, during the discharge period (afternoon to evening), NAPW releases stored heat faster into the basin water, leading to its relatively lower temperature compared to PW. Moreover, the figure indicates that the maximum recorded temperature of NAPW exceeds that of PW, which can be explained by the variation in their specific heat capacities, as summarized in Table 5.

**Table 5:** Thermo-physical properties of the paraffin wax (PW), Al_2_O_3_-PW nano composite (NAPW) and aluminum oxide (Al_2_O_3_)^50,54,55,59^.

Property	Storage medium				
	PW	NAPW			Al_2_O_3_
		0.1wt%	0.2wt%	0.3wt%	
Peak melting temperature, °C	57.23	56.8	56.3	55.8	–
Peak solidification temperature, °C	48.38	48.2	48.1	48	–
Specific heat capacity, J/ (kg. °C)	2000	1994.6	1989.2	1983.9	773
Density in solid state, kg/m^3^	880	883	886	889	3880
Density in liquid state, kg/m^3^	760	763.12	766.24	769.36	–
Thermal conductivity in both solid and liquid phases, W/(m.°C	0.2	0.20314	0.20628	0.20942	40
Fusion latent heat, kJ/kg	90.7	90.301	89.905	89.512	–
Solidification latent heat, kJ/kg	78.2	77.802	77.408	77.016	–

**Fig. 12:**
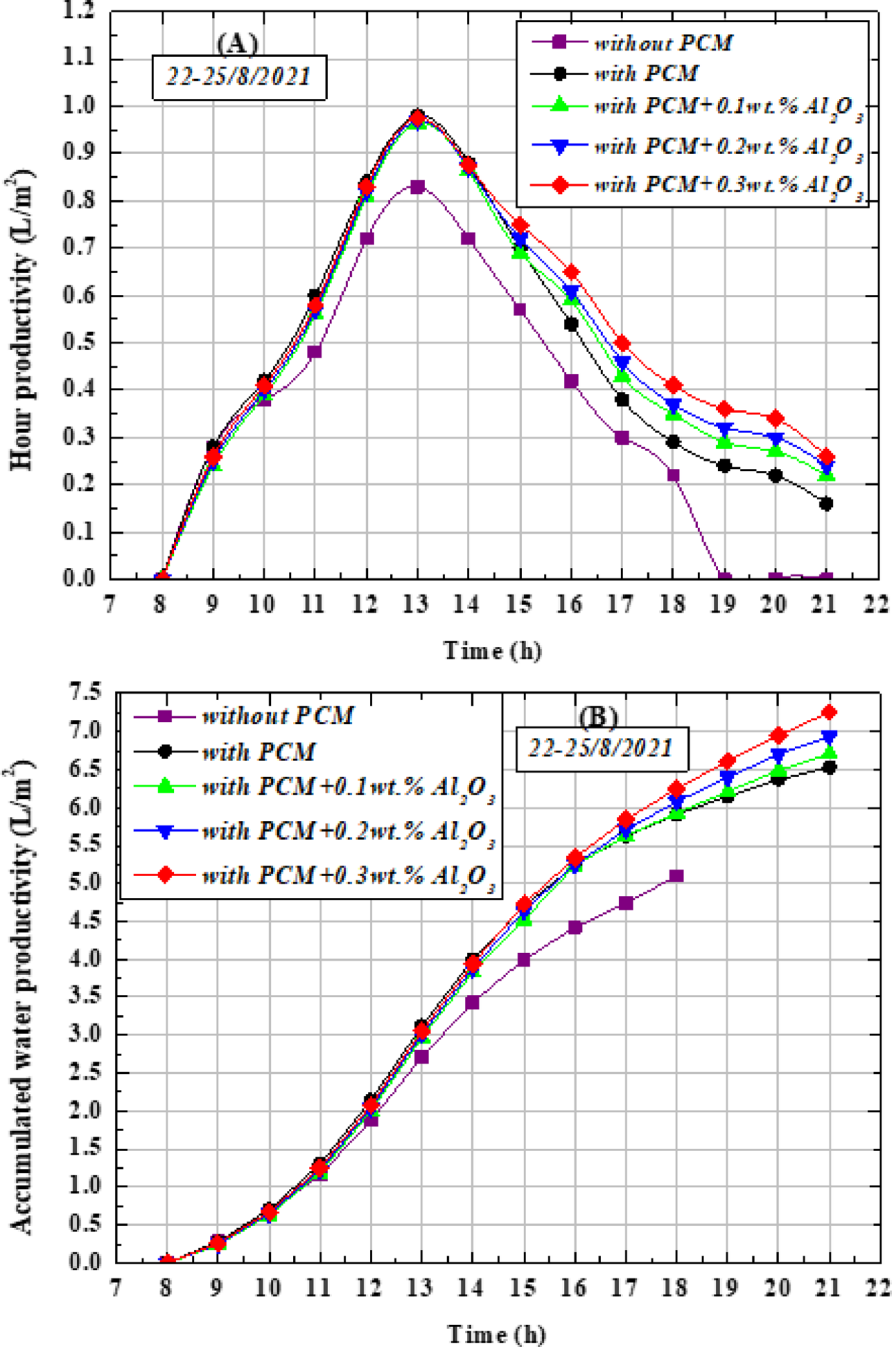
Performance of the OTSS with PW, without PW, and with NAPW (**A**) Hour productivity (**B**) Accumulated water productivity.

Figure 12(A) illustrates the hourly productivity of freshwater for the present study cases without PW, with PW and with NAPW with different volume concentrations of Al_2_O_3_ (0.1, 0.2, and 0.3)wt.% where the figure shows that the productivity increases gradually in all cases From zero in the morning at 8 AM until it reaches its maximum value at 1 PM and then returns to decrease gradually until it reaches its lowest value at 9 PM at the end of the day. The maximum hourly production rate of NAPW with different size concentrations of Al_2_O_3_ 0.1, 0.2, and 0.3wt.% is 0.955, 0.96, and 0.97 L/m^2^/h, respectively, while with pure PW it was 0.98 L/m^2^/h and its value was without PW L/m^2^/h 0.84 at 1PM. Figure 12(B) shows the total freshwater produced in all cases that were implemented on OTSS, where the Accumulated water productivity without PW and with PW amounted to 5.1 and 6.53 L/m^2^/day, respectively, but after using NAPW with different size concentrations of Al_2_O_3_ 0.1, 0.2, and 0.3wt.% the productivity increased to 6.71, 6.94, and 7.26 L/m^2^/day respectively, and therefore the best concentration of Al_2_O_3_ nanoparticles in Paraffin wax is ɸ = 0.3wt%, which achieved the highest system productivity and its value is 7.26 L/m^2^/day, meaning that there is an improvement in the total productivity by 28.03, and 42.35% when using pure PW and NAPW at a concentration of 0.3wt% Al_2_O_3_, respectively, compared to the first case without PW, in addition, the productivity was improved by using NAPW by 11.17% compared to pure PW, which indicates the high heat capacity and high thermal conductivity of NAPW.

As noticed in Fig. 12(B), there is no significant difference in productivity in the two cases using pure PW and using NAPW in the first half of the day due to the absorption and storage of solar radiation needed for the melting process, but after 2 PM the productivity increases with NAPW due to the increased heat capacity of NAPW and the stored heat that is discharged and released to the saltwater basin faster compared to pure PW in terms of absorption and faster release of thermal energy and this is suitable for storing solar energy^59,60^. In addition, the dispersion of Al_2_O_3_ nanoparticles in paraffin wax increases thermal diffusion, faster water heating, and improves the physical bonding interaction between paraffin wax molecules to overcome the thermal resistance during the heat transfer process^59^. The addition of Al_2_O_3_ nanoparticles to paraffin wax lead to a decrease in the melting temperature from 57.23 °C to 55.8 °C at a concentration of 0.3wt% and an increase in system efficiency by 12.27% compared to pure PW as well as an increase in operating hours from 6 to 9 pm.

### Comparison with previous work

**Fig. 13:**
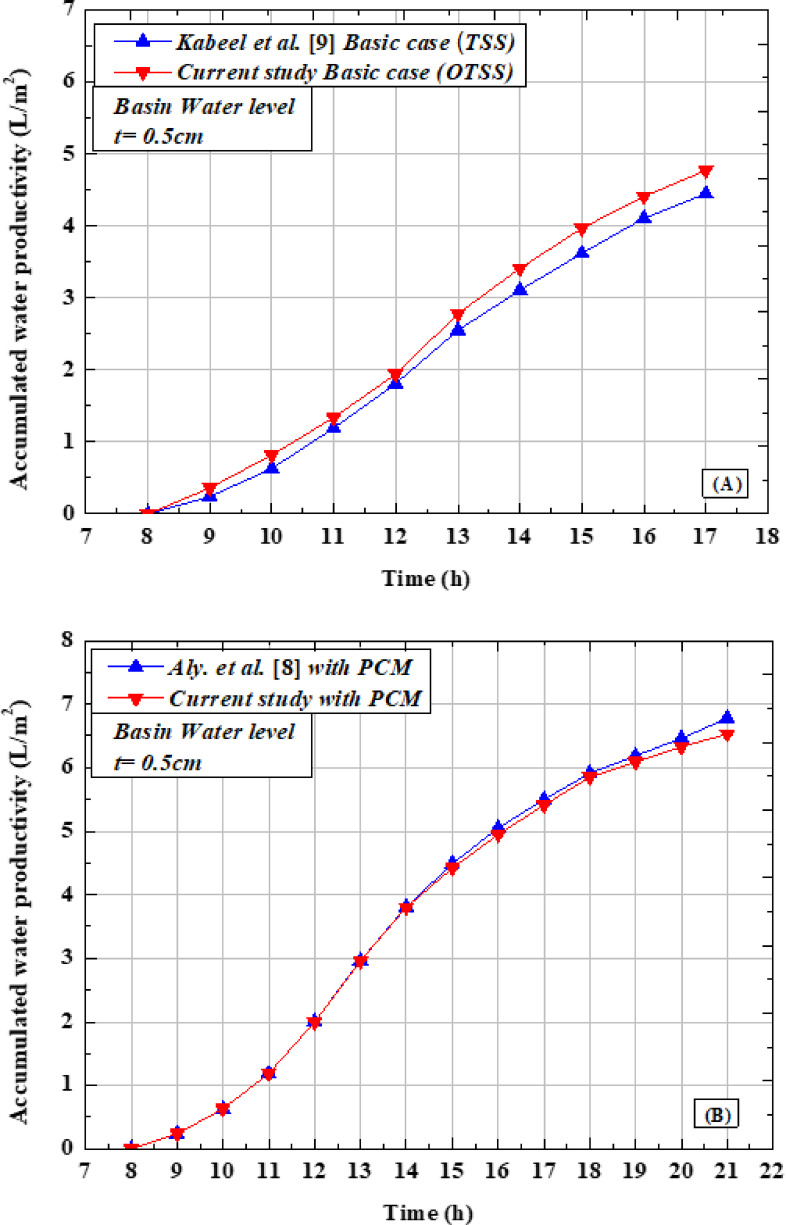

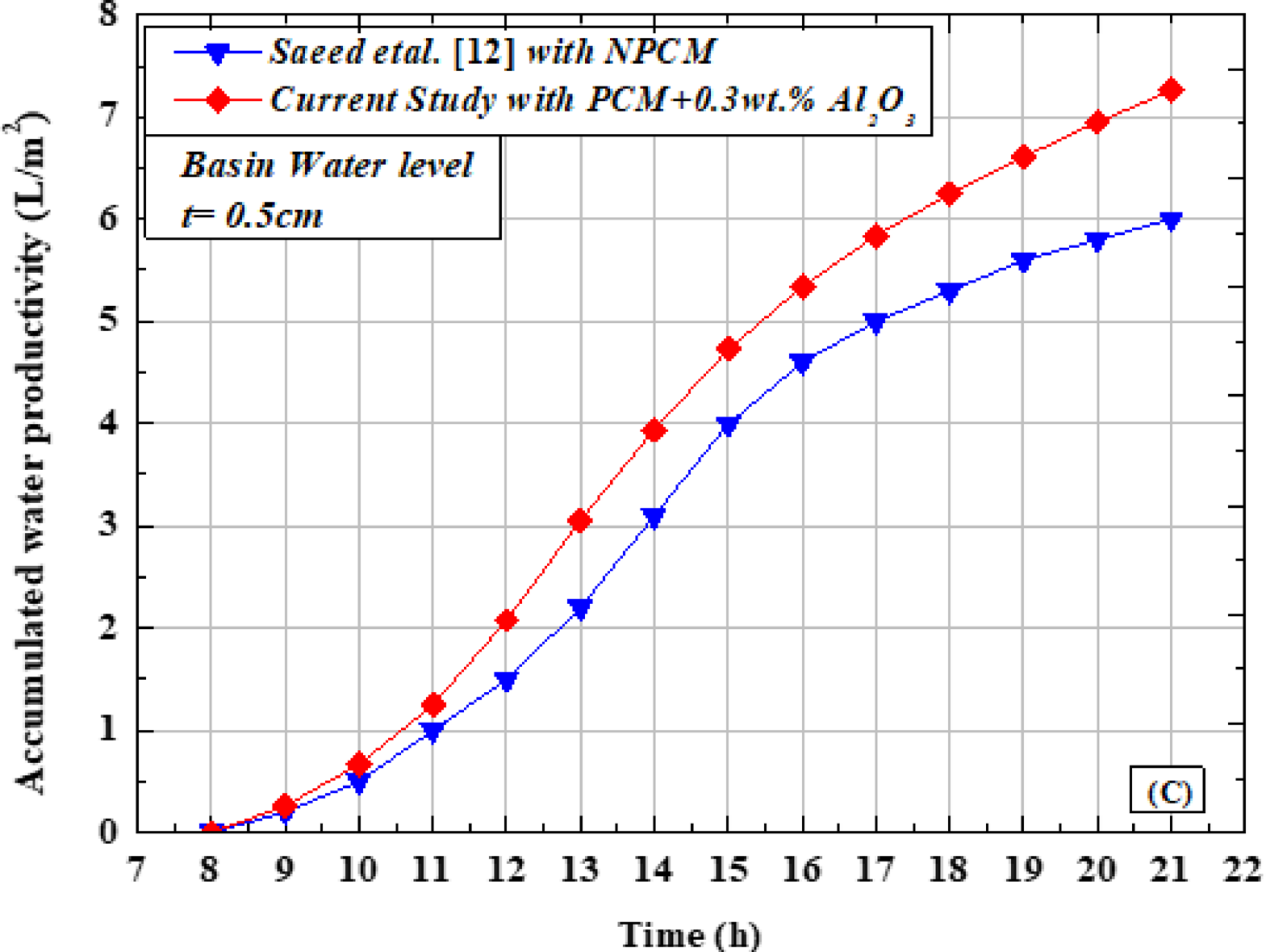
Variation of the current study accumulated water productivity and the results of the open published results, (**A**) OTSS basic case, (**B**) OTSS with PCM case, and (**C**) OTSS with NAPW (PCM + 0.3 wt. % Al2O3).

Figures 13(A–C) compare the results of the present study with the results of *Kabeel* et al.^9^. It can be noticed from Fig. 13A that the accumulated water productivity recorded was 4.77 L/m^2^·day compared to 4.45 L/m^2^·day that recorded by Kabeel et al.^9^ at the same basin water level Fig. 13A. Figure 13B illustrates the results of the present study with PCM with the results of Aly et al.^9^. It can be noticed from the figure that the accumulated water productivity using PCM recorded was 6.78 L/m^2^·day compared to 6.53L/m^2^·day that recorded by Aly et al.^9^ at the same basin water level. Figure 13C presents the results of the present study with NPCM (PCM + 0.3wt. % Al_2_O_3_) with the results of Saeed et al.^12^. It can be noticed from the figure that the accumulated water productivity using NPCM recorded was 7.26L/m^2^·day compared to 6 L/m^2^·day that recorded by Saeed et al.^9^ at the same basin water level of 0.5cm.

### The OTSS thermal efficiency

The thermal efficiency (${ƞ}_{d}$) of the OTSS is determined using Eq. (2). It represents the ratio of the useful energy gained, expressed as the product of the hourly freshwater yield and the latent heat of vaporization of water, to the total solar energy input, which is obtained from the daily accumulated solar radiation intensity multiplied by the absorber surface area^20,61–63^.

2 $$\eta _{d} =\frac{\sum {m}_{dis }{\times h}_{fg }}{\sum I\left(t\right) \times { A}_{abs }\times 3600}$$

Where:

${m}_{dis}$ is the hourly mass of freshwater produced (kg/h),

$I(t)$ represents the daily solar irradiance (W/m^2^),

${A}_{abs}$ is the absorber surface area (m^2^), and.

${h}_{fg}$ denotes the latent heat of vaporization of water (J/kg).

The vaporization latent heat of the water was mentioned in references^16,19^, and to find it, the following equation is used and it is calculated at the average temperature of the water in the basin (${T}_{bw}$).

3 $${\mathrm{h}}_{\text{fg }}={10}^{3}\times \left[2501.9-2.40706\times {\mathrm{T}}_{\mathrm{bw}}+1.192217\times {10}^{-3}\times {{\mathrm{T}}_{\mathrm{bw}}}^{2}-1.5863\times {10}^{-5 }\times {{\mathrm{T}}_{\mathrm{bw}}}^{3} \right]$$

The result of Eq. (3) is in J / Kg.

**Fig. 14:**
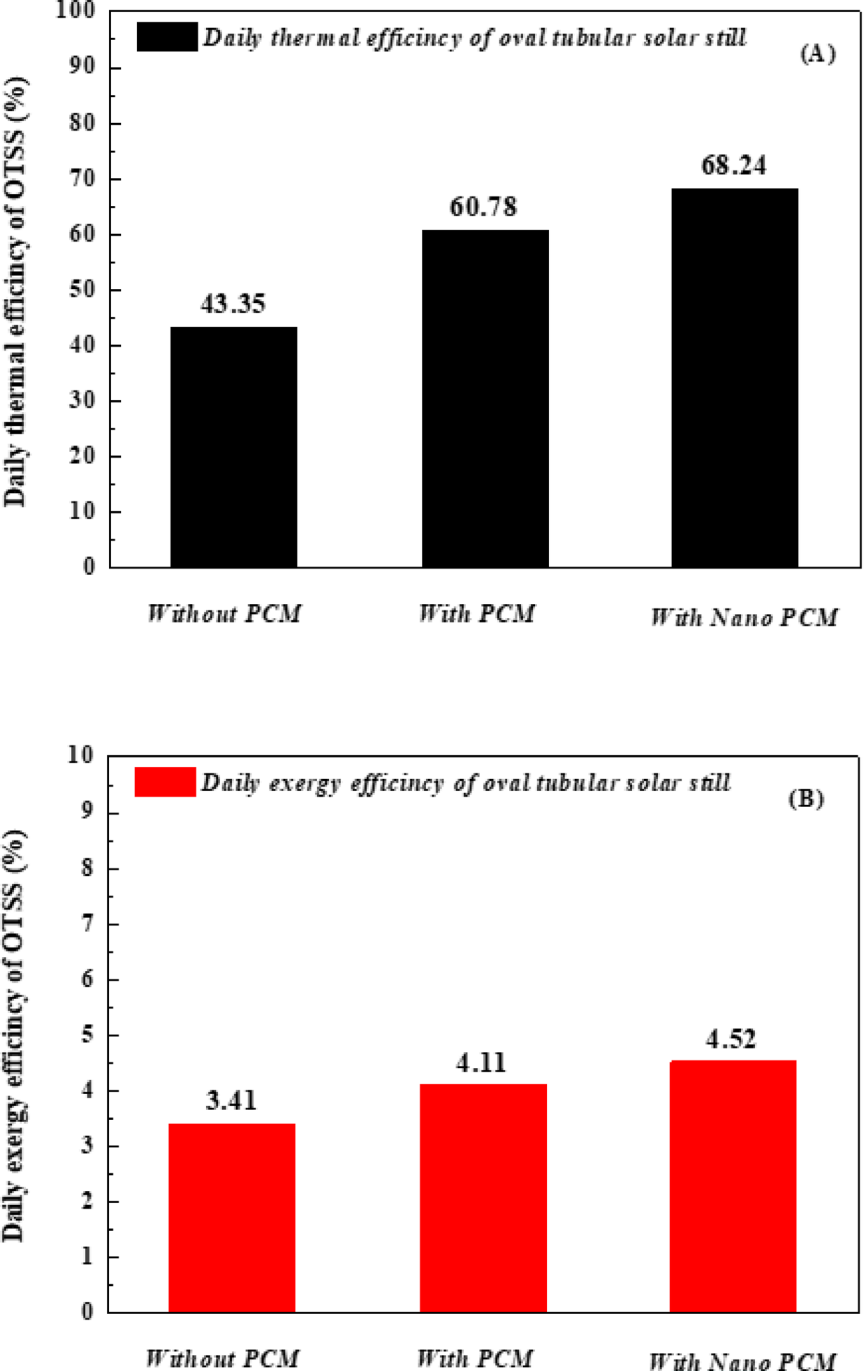
Comparative study of OTSS with PCM, without PCM, and with NPCM (**A**) Thermal efficiency (**B**) Exergy efficiency.

Based on the results obtained and their substitution into Eq. (2), the OTSS exhibited a thermal efficiency of 43.35% at the lowest water depth of 0.5 cm without any system enhancements. When pure paraffin wax (PW) was introduced, the efficiency increased to 60.78%. Furthermore, the incorporation of Al_2_O_3_ nanoparticles into paraffin wax (NAPW) further enhanced the efficiency, reaching 68.24% at a nanoparticle concentration of 0.3 wt.% Fig. 14 (A). This demonstrates that the efficiency improved by 40.20% with PW and by 57.41% with NAPW compared to the baseline case without PW. Moreover, the use of NAPW resulted in an additional improvement of 12.27% relative to pure PW. A summary of these findings is presented in Table 5.

### Exergy efficiency of OTSS

Exergy analysis is used to determine the energy quality for thermal systems and symbolizes the exergy efficiency of solar still with the symbol ($\eta _{{Ex}}$), and it is defined as the output to the input exergy ratio of the system^61,64^.

4 $$\eta _{Ex} =\frac{Output\, Exergy}{Input\, Exergy}= \frac{{E}_{x evap}}{{E}_{x input}}$$

The system energy output ${E}_{x output}$ is expressed as a function of the amount of desalinated water collected from the distillate, and it can be calculated using the following equation^65,66^:

5 $${E}_{x output}={E}_{x evap}=\frac{{m}_{dis }\times { h}_{fg }}{\left( 3600s\right)}\times \left(1-\frac{{T}_{a}+273.15}{{T}_{bw}+273.15}\right)$$

Knowing that (${m}_{dis}$ ) total freshwater produced per hour Kg/h, (${h}_{fg}$) latent heat of vaporization of water J/kg, (${T}_{bw}$ ) basin water temperature °C, and (${T}_{a}$ ) ambient temperature °C. The system exergy input (${\mathrm{E}}_{x sun}$) is defined as^64^:

6 $${E}_{\text{x sun}}= {\text{ A}}_{\text{abs }}\times {\text{ I}\left(\mathrm{t}\right)}_{\text{s }}\left[1-\frac{4}{3}\times \left(\frac{{\mathrm{T}}_{\mathrm{a}}+273.15}{{\mathrm{T}}_{\mathrm{s}}}\right)+\frac{1}{3}\times {\left(\frac{{\mathrm{T}}_{\mathrm{a}}+273.15}{{\mathrm{T}}_{\mathrm{s}}}\right)}^{4}\right]$$

where ($I(t)$) is the total solar intensity/day W/m^2^, (${A}_{abs}$) is the overall area of absorption m2, (${T}_{a}$ ) ambient temperature °C, and (${T}_{s}$) is the sun temperature °C.

The thermal exergy efficiency without PW, with PW, and with NAPW was 3.41, 4.11, and 4.52%. This indicates that the exergy efficiency has improved by 20.52% with PW, and it has improved by 32.55% with NAPW compared to the first case without PW, in addition, the Thermal efficiency was improved by using NAPW by 9.97% compared to pure PW. The results of the study may be summarized in Table 6

**Table 6:** Comparison between the present work results of the OTSS Without additions, with PW, and with NAPW.

System/comparison parameter	OTSS Basic case	OTSS With PW	OTSS NAPW
Maximum productivity/hour (L/m^2^/h)	0.84	0.98	0.97
Accumulated water productivity (L/m^2^/day)	5.11	6.53	7.26
Thermal efficiency ( % )	43.35	60.78	68.24
Improvement ratio in thermal efficiency ( % )	–	40.20	57.41
Exergy efficiency( % )	3.41	4.11	4.52
Improvement ratio in exergy efficiency ( % )	–	20.52	32.55
Cost per liter ( $/ L)	0.0208	0.0175	0.0163

## Economic analysis

The economic feasibility of the oval tubular solar still (OTSS) was evaluated by estimating the cost per liter (CPL) of freshwater produced over the system lifetime. The fixed cost of the OTSS components is listed in Table 7.

**Table 7:** Cost of the elements of OTSS ($).

Elements	Without PW Basic case	With PW	With NAPW
Transparent oval tube	105	105	105
Black saltwater basin	25	25	25
Water ducts, valves, and paint	5	10	10
Silicone and adhesives	10	10	10
Paraffin wax	–	5	5
Paraffin basin	–	15	15
Aluminum oxide nano particles	–	–	3
Manufacture	100	105	105
Total fixed cost (F)	245	275	278

To account for the time value of money, the capital recovery factor (CRF) was used to convert the initial capital investment into an equivalent uniform annual cost. The CRF is expressed as:

7 $$CRF = \frac{{i(1 + i)^{n} }}{{(1 + i)^{n} - 1}}$$

where i is the annual interest rate and n is the system lifetime (years). In this study, the lifetime of the OTSS is assumed to be 10 years.

The annualized fixed cost (AFC) is calculated using:

8 $${\mathrm{AFC}}\, = \,{\mathrm{F}}\, \times \,{\mathrm{CRF}}$$

where F represents the total fixed capital cost.

In addition to the capital cost, the system incurs annual variable costs. According to previous studies^20^, the annual variable cost is assumed to be 30% of the fixed cost. Therefore, the annual variable cost (AVC) can be written as:

9 $${\mathrm{AVC}}\, = \,0.{\mathrm{3F}}$$

The capital fixed factor (CFF), which represents the ratio of the total annualized cost to the fixed cost, can therefore be expressed as:

10 $${\mathrm{CFF}}\, = \,{\mathrm{CRF}}\, + \,0.{3}$$

Accordingly, the total annual cost (TAC) of the system becomes:

11 $${\mathrm{TAC}}\, = \,{\mathrm{F}}\, \times \,{\mathrm{CFF}}$$

The yearly freshwater productivity is calculated as^20^:

12 $${\text{Yearly productivity }}\left( {{\mathrm{TFP}}} \right)\, = \,\left( {\text{Operating days}} \right)\, \times \,{\mathrm{m}}$$

where m is the average daily freshwater yield and the number of operating days per year is assumed to be 340 days, corresponding to the annual sunshine conditions in Egypt.

The cost per liter (CPL) of freshwater is finally determined as^20,32,67^:

13 $${\mathrm{CPL}}\, = \,{\text{TAC }}/{\text{ Yearly productivity}}$$

The economic results for the three investigated cases are summarized in Table 8. The cost per liter was found to be 0.0208 $ without PW, 0.0175 $ with PW, and 0.0163 $ with NAPW. The NAPW configuration represents the most economically favorable case due to its enhanced freshwater productivity and overall system performance.

**Table 8:** OTSS economic analysis.

Items	OTSS without PW (Basic case)	OTSS with PW	OTSS with NAPW
Total cost (TAC), $	318.5	357.5	361.4
Average desalinated water, L/m^2^/day	4.5	6	6.5
TFP , L	15,300	20,400	22,100
Cost per liter, $	0.0208	0.0175	0.0163

The total freshwater production, TFP, throughout the expected lifetime of the OTSS is determined using the following equation:

14 $${\mathrm{TFP}}\, = \,{\text{Yearly productivity}}\, \times \,{\mathrm{n}}$$

Where (n) is the number of assumed years expected for the still which is assumed to be 10 years^20^.

Table 9 compares the present results with previously published studies on OTSS, TSS, and conventional solar stills (CSS). The findings indicate that the proposed OTSS achieves a lower cost per liter while maintaining superior productivity and efficiency compared with the reported systems.

**Table 9:** Comparison of present OTSS performance and different CSS and TSS.

References	Total freshwater (L/m^2^/day)	$$\eta _{d}$$, (%)	Cost per liter, ($)
Kabeel et al. ^9^	5.45	41.4	0.023
Kabeel et al. ^14^	2.1	29.20	0.0359
Kabeel et al. ^31^	2	29.98	–
Omara et al. ^68^	–	29.98	0.0449
Suneesh et al. ^69^	–	–	0.042
Winston et al. ^70^	–	29.98	0.200
Present study (OTSS)	5.11 without PW	43.35	0.0208
	6.53 with PW	60.78	0.0175
	7.27 with NAPW	68.24	0.0163

## Conclusions

In this study, a novel design of solar stills, named OTSS, was presented and investigated under three conditions: without paraffin wax (PW), with PW, and with PW enhanced by Al_2_O_3_ nanoparticles (NAPW), under Cairo, Egypt climatic conditions. Several factors affecting system performance were examined, including saline water depth (0.5, 1, 1.5, and 2 cm), the use of PW, and different Al_2_O_3_ concentrations (0.1, 0.2, and 0.3 wt.%).

The key findings can be summarized as follows:

- Without PW, system productivity decreased with increasing water depth, achieving a maximum yield of 5.11 L/m^2^/day at 0.5 cm depth.
- Using PW at the same depth increased the maximum production to 6.53 L/m^2^/day.
- The optimal Al_2_O_3_ concentration in paraffin wax was 0.3 wt.%, where the NAPW-enhanced system achieved the highest productivity of 7.26 L/m^2^/day at 0.5 cm depth.
- Total productivity improved by 28.03% and 42.35% using PW and NAPW, respectively, compared to the system without PW, with an additional 11.17% improvement when using NAPW over pure PW.
- The daily thermal efficiency at 0.5 cm depth was 43.35% without PW, 60.78% with PW, and 68.24% with NAPW, showing improvements of 40.20% and 57.41% for PW and NAPW, respectively, over the baseline, and an additional 12.27% increase for NAPW compared to PW.
- Daily exergy efficiency was 3.41% without PW, 4.11% with PW, and 4.52% with NAPW, reflecting enhancements of 20.52% and 32.55%, respectively, with an additional 9.97% improvement for NAPW over PW.
- Incorporation of Al_2_O_3_ nanoparticles into PW improved thermal storage capacity, thermal conductivity, and accelerated the melting process, resulting in a higher peak temperature for NAPW compared to pure PW.
- The cost per liter of freshwater production was $0.0208 without PW, $0.0175 with PW, and $0.0163 with NAPW.
- It can be concluded that using NAPW represents the most favorable approach**,** providing the highest system efficiency and productivity while reducing water production costs. These results demonstrate the promising potential of nanomaterial-enhanced paraffin wax to improve the economic and thermal performance of solar still systems.

## Future scope

It is recommended to utilize nanoparticles with different materials and concentrations to enhance the thermal conductivity of phase change material and increase the productivity of the OTSS. Also, different PCM masses/thicknesses and oval tube aspect ratios need to be investigated.

## Data Availability

The datasets generated during and/or analyzed during the current study are available from the corresponding author upon reasonable request.

## List of symbols

$${\mathrm{A}}_{\mathrm{abs}}$$: Absorption total area, m^2^
$$\mathrm{A}$$: Accuracy
C: Total cost, $
CPL: Cost per liter, $
Ex: Exergy, J
F: Fixed cost, $
$${h}_{fg}$$: Vaporization latent heat of, J/kg
$${I}_{d}$$: Total daily solar intensity, W/m^2^
$$I(t)$$: Total solar intensity, W/m^2^
$${m}_{dis}$$: Total freshwater rate, kg/h
n: OTSS life time
$${P}_{d}$$: Daily productivity, L/m^2^/ day
t: Temperature, °C
$${T}_{a}$$: Ambient air temperature, °C
$${T}_{bw}$$: Temperature of the water in basin, °C
TFP: Total freshwater produced, L
$${T}_{s}$$: Sun temperature, °C
V: Variable cost, $

## Greek letters

$$\eta _{d}$$: Thermal efficiency, %
$$\eta _{Ex}$$: Exergy efficiency, %
ω: Uncertainty

## Scripts

A: Ambient
bw: Water in the basin
W: Wind
X: Variable
xn: Parameter of interest

## Acronyms and abbreviations

OTSS: Oval tubular solar still,
PCM: Phase change materials,
PSS: Pyramid solar still,
PW: Paraffin wax
NAPW: Nano alumina paraffin wax
TSS: Tubular solar still
CSS: Conventional solar still
